# Development of Multiplex Assays for the Identification of Zoonotic *Babesia* Species

**DOI:** 10.3390/pathogens13121094

**Published:** 2024-12-11

**Authors:** Ana Cláudia Calchi, Charlotte O. Moore, Lillianne Bartone, Emily Kingston, Marcos Rogério André, Edward B. Breitschwerdt, Ricardo G. Maggi

**Affiliations:** 1 Vector-Borne Bioagents Laboratory (VBBL), Department of Pathology, Reproduction and One Health, School of Agricultural and Veterinarian Sciences (FCAV), São Paulo State University (UNESP), Jaboticabal CEP 14884-900, Brazil; ana.calchi@unesp.br (A.C.C.); mr.andre@unesp.br (M.R.A.); 2 Intracellular Pathogens Research Laboratory, Comparative Medicine Institute, College of Veterinary Medicine, North Carolina State University, Raleigh, NC 27606, USA; comanvel@ncsu.edu (C.O.M.); lily_bartone@ncsu.edu (L.B.); emkingst@ncsu.edu (E.K.); ebbreits@ncsu.edu (E.B.B.)

**Keywords:** babesiosis, diagnosis, zoonotic *Babesia*, tick-borne diseases

## Abstract

More than one-hundred *Babesia* species that affect animals and humans have been described, eight of which have been associated with emerging and underdiagnosed zoonoses. Most diagnostic studies in humans have used serology or molecular assays based on the 18S rRNA gene. Because the 18S rRNA gene is highly conserved, obtaining an accurate diagnosis at the species level is difficult, particularly when the amplified DNA fragment is small. Also, due to its low copy number, sequencing of the product is often unsuccessful. In contrast, because the *Babesia* internal transcribed regions (ITS), between 18S rRNA and 5.8S rRNA, and between 5.8S rRNA and 28S rRNA, contain highly variable non-coding regions, the sequences in these regions provide a good option for developing molecular assays that facilitate differentiation at the species level. In this study, the complete ITS1 and ITS2 intergenic regions of different Piroplasmida species were sequenced to add to the existing GenBank database. Subsequently, ITS1 and ITS2 sequences were used to develop species-specific PCR assays and specific single-plex and multiplex conventional (c)PCR, quantitative real-time (q)PCR, and digital (d)PCR assays for four zoonotic *Babesia* species (*Babesia divergens*, *Babesia odocoilei*, *Babesia duncani*, and *Babesia microti*). The efficacy of the assay protocols was confirmed by testing DNA samples extracted from human blood or enrichment blood cultures. Primers were first designed based on the 18S rRNA-5.8S rRNA and 5.8S rRNA-28S rRNA regions to obtain the ITS1 and ITS2 sequences derived from different Piroplasmida species (*B. odocoilei*, *Babesia vulpes*, *Babesia canis*, *Babesia vogeli*, *Babesia gibsoni*, *Babesia lengau*, *Babesia divergens*-like, *B. duncani*, *B. microti*, *Babesia capreoli*, *Babesia negevi*, *Babesia conradae*, *Theileria bicornis*, and *Cytauxzoon felis*). Subsequently, using these sequences, single-plex or multiplex protocols were optimized targeting the ITS1 region of *B. divergens*, *B. microti*, and *B. odocoilei*. Each protocol proved to be sensitive and specific for the four targeted *Babesia* sp., detecting 10^−2^ (for *B. microti* and *B. odocoilei*) and 10^−1^ (for *B. divergens* and *B. duncani*) DNA copies per microliter. There was no cross-amplification among the *Babesia* species tested. Using 226 DNA extractions from blood or enrichment blood cultures obtained from 82 humans, *B. divergens* (seven individuals), *B. odocoilei* (seven individuals), and *B. microti* (two individuals) were detected and identified as a single infection, whereas co-infection with more than one *Babesia* sp. was documented by DNA sequencing in six (7.3%) additional individuals (representing a 26.8% overall prevalence). These newly developed protocols proved to be effective in detecting DNA of four *Babesia* species and facilitated documentation of co-infection with more than one *Babesia* sp. in the same individual.

## 1. Introduction

Piroplasmids, apicomplexan protozoa transmitted by Ixodid ticks, can cause diseases in animals and humans. These protozoa form a polyphyletic group comprising four genera: *Babesia*, *Theileria*, *Cytauxzoon*, and *Rangelia* [1,2,3,4]. Currently, there are more than 100 *Babesia* (Phylum: Apicomplexa; Order: Piroplasmida) species that infect domestic, wildlife, and human hosts, with several new species and genotypes being identified every year [2,5,6,7,8,9,10]. Because of the worldwide economic relevance (especially affecting production animals) and due to the increasing impact on human health, the genus *Babesia* is of medical concern, particularly as an emerging zoonotic pathogen [1,11,12,13,14,15]. Human *Babesia* sp. transmission has been predominantly associated with tick bites [2], with *Ixodes scapularis* being the primary tick vector in the USA and Canada [14,15,16,17,18,19,20,21,22,23,24] and *Ixodes ricinus* and *Ixodes canisuga* being the primary vectors in Europe [25,26]. Other modes of transmission such as transfusion of *Babesia*-contaminated blood, organ transplantation, and oral and transplacental transmission have also been reported [27,28,29,30,31,32,33,34,35].

From over one-hundred *Babesia* species recognized to date, only eight species, confirmed by DNA sequencing, have been reported to cause infections in humans (Table S1): *Babesia bigemina*, *Babesia crassa* (including *B. crassa*-like), *Babesia divergens* (including *B. divergens*-like MO1) *, Babesia duncani*, *Babesia microti*, *Babesia motasi*, *Babesia odocoilei*, and *Babesia venatorum* [1,5,12,14,15,27,28,36,37,38,39,40,41,42,43,44,45,46,47,48,49,50,51,52,53,54,55,56,57,58,59,60,61,62,63,64,65,66,67,68,69]. Despite being reported as a worldwide infection in people [15,53], 95% of all human babesiosis cases have been reported from the USA and Canada [38,70].

Babesiosis symptoms in people vary from asymptomatic, to non-specific, to life-threatening hemolytic anemia, particularly in splenectomized or otherwise immunocompromised individuals [27,39,40,41,71,72]. Predominant symptoms and clinical findings include myalgia, fever, sweating, chills, profound fatigue, sleep disorders, and hepatosplenomegaly. Hemolytic anemia severity can be related to immunosuppressive factors, such as splenectomy and comorbidities (e.g., cancer, chronic heart, lung, renal, or liver disease, infection with HIV/AIDS, or patients receiving immunosuppressive drugs). Illness typically occurs after an incubation period of 1 to 4 weeks following tick transmission and can persist for several weeks prior to diagnosis and treatment [27,39,40,41,71,72].

Historically, diagnosis of animal and human babesiosis was primarily based on microscopical observation of intraerythrocytic *Babesia* merozoites/trophozoites in stained blood smears and serology. Unfortunately, merozoite/trophozoite visualization has poor sensitivity due to the low number of circulating infected erythrocytes. For example, less than 1% of erythrocytes can be infected with *B. microti* during the acute disease and chronic illness phase of human babesiosis. [2]. Serology does not confirm ongoing infection, and does not definitively determine the infecting species due to a high degree of cross-reactivity among closely related *Babesia* species (i.e., *B. divergens, B. odocoilei*, and *B. duncani*) [39,69]. Molecular methods, most often PCR targeting the highly conserved 18S rRNA gene, followed by DNA sequence analyses, have been a widely used method for *Babesia* sp. detection in clinical cases, which has facilitated species identification and, importantly, phylogenetic inferences at the taxon level (Table S1). In recent years, due to highly conserved 18S rRNA *Babesia* sequences (that in some instances impair speciation by DNA sequencing) and because the internal transcribed spacer (ITS) between the 18S rRNA, 5.8S rRNA, and 28S rRNA genes are more highly variable, the ITS regions have been increasingly used for the detection and identification of specific *Babesia* sp., such as *B. divergens*, *B. microti*, and *B. odocoilei* in people, as well as for different *Babesia* spp. infecting animals [1,2,37,42,43,73,74,75].

Because the regions between 18S rRNA and 5.8S rRNA (referred to as *Babesia* ITS1 in this manuscript) and the 5.8S rRNA and 28S rRNA genes (referred to as *Babesia* ITS2) had higher discriminatory power (when compared with amplification targets derived from the conserved 18S rRNA region), we developed conventional (cPCR), quantitative real-time (qPCR), and digital (dPCR) PCR assays for detecting and identifying *Babesia* at both genus and species levels (specifically aimed at *B. divergens*, *B. duncani*, *B. microti*, and *B. odocoilei*, the predominant or suspected *Babesia* spp. that infect humans in the USA [15,39,76,77,78]).

## 2. Material and Methods

### 2.1. Animal and Human Sample Sources

*Babesia*-infected animal samples were kindly provided by Dr. Barbara Qurollo and Brittany Thomas from the Vector-Borne Disease Diagnostic Laboratory, College of Veterinary Medicine [79], North Carolina State University, and from Dr. Sam Telford, Cummings School of Veterinary Medicine, Tufts University.

All human study participants provided three blood and serum specimens collected within a 7-day period. These individuals were previously tested because of a history of arthropod or animal contact as a component of an Institutional Review Board (IRB)-approved study entitled “Detection of *Bartonella* Species in the Blood of People with Extensive Animal Contact” (North Carolina State University Institutional Review Board, IRB#s 4925-03 and 164-08-05). The culture and molecular testing approach used in this study was described in prior publications from our research group [1,80,81,82]. A total of 226 human DNA blood and enrichment blood culture samples from 82 patients from 7 countries (including 27 USA states) were tested for the presence of *Babesia* species DNA.

### 2.2. GenBank Reference Sequences Used in This Study

The following 18S rRNA, 5.8S rRNA, and 28S rRNA gene reference sequences from representative *Babesia* species were retrieved from the GenBank database (http://www.ncbi.nlm.nih.gov, accessed on 1 October 2024). Using Clustal W multi-sequence alignment (AlignX, Vector NTI Advanced 10.3.0 from Invitrogen, Waltham, MA, USA), the complete or partial regions between 18S and 28S rRNA from *B. bigemina* (HQ840960), *B. canis* (AY072926), *B. vogeli* (HQ148664), *Babesia* sp. Coco (AY618928, EU109720, EU109721), *B. conradae* (AF158702), *B. crassa* (MK240324, AY260176, KX590751), *B. divergens* (AY572456, EU182599, EU185801, EF458187, EU182603, EU182598), *B. duncani* (HQ289870, MH333111), *B. gibsoni* (CP141526), *B. microti* (AB112337, GU230755, LN871598, AB190435, MK609547, XR_002459986), *B. motasi* (AY260179), *B. odocoilei* (AF158711, AY339747, AY339754, AY339756, AY339757, AY339759, AY345122, BOU16369, KC1622888, MF357056), *B. orientalis* (HQ840969), *B. poelea* (DQ200887), *B. rodhaini* (AF510201), *B. uriae* (FJ17705), and *B. venatorum* (GQ888709, MG344777, AY046575, KF724377, OP522105) were used as species reference genes for alignments.

### 2.3. Sequence Analysis, Babesia Genus ITS1 and ITS2 Region Primer Design, DNA Amplification, and Sequence Analysis of Different Babesia Species from Naturally Infected Animals

**Figure 1:**
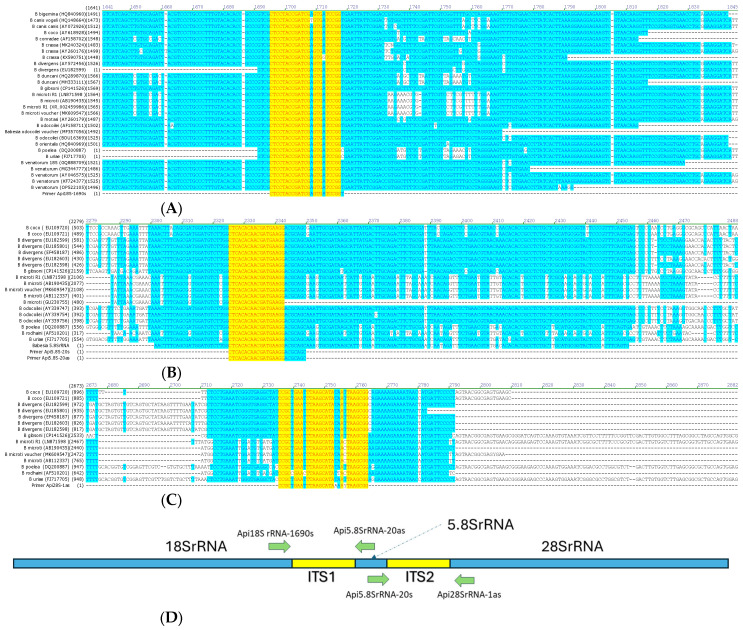
(**A**): Region in yellow of the conserved downstream 18S rRNA for primer Api18S-1690s. (**B**): Region in yellow of the conserved downstream 5.8S rRNA for primer Api5.8S-20s and Api5.8S-20as. (**C**): Region in yellow of the conserved downstream 28S rRNA for primer Api28S-1as. (**D**): Schematic diagram of the intergenic transcribed spacer 1 (ITS1) and 2 (ITS2) DNA regions targeted for amplification and sequencing of several *Babesia* species.

After DNA sequence alignment and homology analysis of GenBank reference genes between 18SrRNA and 5.8S rRNA, and between 5.8SrRNA and 28SrRNA (Figure 1A–D), oligonucleotides were designed: Api18S rRNA-1690s: 5′ CTCCTACCGATCGAGTGATCCGGT 3′ (selected from a conserved region at the 3′ end of the *Babesia* 18S rRNA gene); Api5.8SrRNA-20as: 5′ GCTGCGTCCTTCATCGTTGTGTGAG 3′; Api5.8SrRNA-20s: 5′ CTCACACAACGATGAAGGACGCAGC 3′ (selected from a conserved region in the *Babesia* 5.8S rRNA gene); and Api28SrRNA-1as: 5′ CCGCTGAATTTAAGCATAAAAYTAAGCGG 3′ (selected from a conserved region at the 5′ end of the *Babesia* 28S rRNA gene). These PCR primers were used for DNA amplification, using cPCR and qPCR, followed by sequencing of the complete intergenic ITS1 and ITS2 regions of several *Babesia* species from infected animals (high-copy-number samples previously characterized by qPCR and dPCR targeting 18S rRNA, [79]), including *B. odocoilei* (from infected caribou [*Rangifer tarandus*]), *Babesia vulpes*, *B. canis*, *B. vogeli* and *Babesia gibsoni* (from infected dogs), *B. lengau* (from an infected cheetah [*Acinonyx jubatus*]), *B. divergens*-like (isolate MO-1 from a infected rabbit [*Sylvilagus* sp.]), *B. duncani* (strain J3 from infected hamster [*Mesocricetus auratus*]), *B. microti* (isolate GI from infected hamster), *Theileria bicornis* (from infected rhinoceros [*Diceros bicornis*]), and *Cytauxzoon felis* (from infected cat) (Table S2). The same conventional and qPCR primers sets were used for the amplification of *Babesia* ITS1 and *Babesia* ITS2 regions from each of the above *Babesia* species.

For conventional cPCR, amplification was performed with a master-mix reaction, at 25 μL final volume per reaction, comprising 7.3 μL of molecular-grade water (QIAGEN Germantown, MD, USA), 12.5 μL of 2× My Taq HS Red Mix (Bioline, Memphis, TN, USA), 0.1 μL of 100 μM each of Api18S rRNA-1690s and Api5.8SrRNA-20as (for *Babesia* ITS1 region) or Api5.8SrRNA-20s and Api28SrRNA-1as (for *Babesia* ITS2 region) as forward and reverse primers (IDT-DNA Technologies, Coralville, IA, USA), and 5μL of extracted DNA. Molecular-grade water and DNA extracted from the blood of a naive dog were used as negative controls. Amplification was performed using a CFX Opus Real-Time PCR System (Bio Rad, Hercules, CA, USA) under the following conditions: 95 °C for 3 min, followed by 45 cycles of denaturing at 94 °C for 10 s, annealing at 66 °C for 15 s, and extension at 72 °C for 20 s. The products obtained in PCR assays were separated by horizontal electrophoresis on 2% agarose gel stained with gelGreen (Thermofisher Scientific, Greenville, NC, USA), visualized under ultraviolet light illumination using a ChemiDoc MP Imaging System (Bio Rad, Hercules, CA, USA) and photographed using Image Lab Software v.3.01 3.01 (Bio Rad, Hercules, CA, USA).

Similarly, real-time qPCR amplification of *Babesia* ITS1 and *Babesia* ITS2 was performed as above with minor modifications: 7.5 μL of molecular-grade water (QIAGEN, Germantown, MD, USA), 12.5 μL of 2× SsoAdvanced SYBR green master mix (Bio Rad, Hercules, CA, USA), 0.2 μL of 100 μM each of oligonucleotide primers Api18S rRNA-1690s and Api5.8SrRNA-20as (for detection of *Babesia* ITS1) and Api5.8SrRNA-20s and Api28SrRNA-1as (for detection of *Babesia* ITS2), and 5μL of extracted DNA. As above, molecular-grade water and DNA extracted from naive dog blood samples were used as PCR negative controls. Amplification was performed in a Bio-Rad CFX 96-well Opus PCR system machine under the following conditions: 95 °C for 3 min, followed by 40 cycles of denaturing at 94 °C for 15 s, annealing at 68 °C for 15 s, and extension at 72 °C for 15 s. An additional gradient cycle from 65 °C to 95 °C was performed for melting curve analysis. Quantification cycle (Cq) values were determined and melting temperature analysis was performed by reading fluorescent signals using the FAM/Syber channel.

For both cPCR and qPCR assays, amplified products were purified and sequenced by Sanger’s method. The obtained sequences were assessed for quality, analyzed using Clustal W multi-sequence alignment (AlignX, Vector NTI Advanced 10.3.0 from Invitrogen), and compared to sequences previously deposited in the GenBank database [1].

### 2.4. Species-Specific qPCR Amplification, Limit of Detection (LOD), and Cross-Amplification Assessment for the Detection of B. microti, B. duncani, B. divergens, and B. odocoilei ITS1 Region

For each representative *Babesia* species (*B. divergens*-like isolate MO-1, *B. duncani* isolate J3, *B. microti* isolate GI, and *B. odocoilei* isolate VB19-09386), the entire *Babesia* ITS1–ITS2 region PCR amplicon obtained using oligonucleotides Api18SrRNA-1690s and Api28SrRNA-1as as forward and reverse primers, respectively, was cloned into plasmid pGEM-T Easy (Promega^®^ Madison, WI, USA), transformed into *E. coli* DH5-α strain-competent cells, and purified using Qiagen plasmid miniprep kits. Plasmid insert DNA was sequenced using M13 primers for species verification and quantified using Qubit Flex Fluorometric Quantification (QIAGEN, Germantown, MD, USA).

Serial dilutions in molecular-grade water, ranging from 10^7^ to 10^−3^ copies/ μL, were used as templates to assess the limit of detection (LOD) by real-time PCR (both using Taqman probes and SYBR Green followed melting curve analysis).

Single-plex species-specific real-time PCR: Real-time PCR was performed using reaction mixture at a 25 μL final volume per reaction. The PCR reaction included 7.3 μL of molecular-grade water (QIAGEN, Germantown, MD, USA), 12.5 μL of 2× SsoAdvanced SYBR green master mix (Bio Rad, Hercules, CA, USA), 0.1 μL of each species-specific primer at 100 μM (IDT-DNA Technologies, Coralville, IA, USA), and 5 μL of DNA sample. Molecular-grade water and DNA extracted from naive dogs and human blood specimens were used as PCR negative controls. Amplification was performed in a Bio-Rad CFX 96-well Opus PCR system machine under the following conditions: 95 °C for 3 min, followed by 40 cycles of denaturing at 94 °C for 10 s, annealing at 68 °C for 10 s, and extension at 72 °C for 10 s. An additional gradient temperature cycle from 65 °C to 95 °C at 0.2 °C per second was performed for melting curve analysis. Quantification cycle (Cq) values were determined and melting temperature analysis was performed by reading fluorescent signals using the FAM/Syber channel. All PCR-positive samples were sequenced by Sanger’s method and analyzed using Clustal W multi-sequence alignment as described above.

### 2.5. Multiplex Species-Specific qPCR

qPCR was performed using the reaction mixture at 25 μL final volume per reaction. PCR amplification included 7.3 μL of molecular-grade water (QIAGEN, Germantown, MD, USA), 12.5 μL of 2× SsoAdvanced probe master mix (Bio Rad, Hercules, CA, USA), 0.1 μL of each species-specific primer at 100 μM (IDT-DNA Technologies Coralville, IA, USA), 0.1 μL of 100 μM (IDT-DNA Technologies Coralville, IA, USA) of each species-specific probe, and 5 μL of DNA sample. Molecular-grade water and DNA extracted from naive dogs and human blood specimens were used as PCR negative controls. Amplification was performed in a Bio-Rad CFX 96-well Opus PCR system machine under the following conditions: 95 °C for 3 min, followed by 40 cycles of denaturing at 94 °C for 10 s, annealing at 68 °C for 10 s, and extension at 72 °C for 10 s. Reading of fluorescent signals for detection of each *Babesia* species was performed during the annealing phase of amplification using FAM (green) for *B. odocoilei*, HEX (yellow) for *B. divergens*, CalFluo590 (red) for *B. microti*, and Cy5 (crimson) for *B. duncani*. Recording of the fluorescent signal quantification value (Cq value) was used to assess positive and negative DNA amplification. As above, all PCR-positive samples were sequenced by Sanger’s method (Genewiz from Azenta, Research Triangle Park, NC, USA) and analyzed using Clustal W multi-sequence alignment (AlignX, Vector NTI Advance 10.3.0 from Invitrogen) to confirm species identification.

## 3. Results

Using Api18S rRNA-1690s and Api5.8SrRNA-20as (as forward and reverse primers), cPCR DNA amplification of the *Babesia* ITS1 region generated a single 557–650 bp band consistent with the expected DNA sizes (depending on species) for all *Babesia* species investigated herein. Importantly, due to a high copy number, all amplicons generated using the *Babesia* ITS1 region assay rendered a high-quality DNA sequence chromatogram, facilitating clear and distinctive differentiation between species and strains (i.e., different *B. odocoilei* strains were identified from different cervid species and from infected humans). Similarly, Api5.8SrRNA-20s and Api28SrRNA-1as (as forward and reverse primers) targeting the *Babesia* ITS2 regions generated a single 385–436 bp band consistent with the expected *Babesia* ITS2 region DNA size (depending on *Babesia* spp.) for *Babesia* species investigated herein (Table S2). Using the qPCR assay method, similar results were obtained for all animal samples tested, generating only a single signal with melting curve temperatures depending on the *Babesia* sp. *Babesia* species ITS1 and ITS2 sequences (amplified by either cPCR or qPCR) were deposited in GenBank (Table S2).

### 3.1. Comparison of Babesia Genus Detection in Human Clinical Samples by qPCR and dPCR Targeting Babesia 18S rRNA Region vs. qPCR Targeting Babesia ITS1 and ITS2 Regions

Previously, our research group described the development of a digital PCR assay targeting the Piroplasmida 18S rRNA region, to detect piroplasmids, at the genus level, in animals and human patients [79]. Despite the higher sensitivity and specificity of the digital PCR assay when compared with real-time PCR targeting the same gene region (including primers and probes) [79], species identification using digital PCR was not possible due to the inability to concentrate target DNA for sequence analysis. Therefore, 226 human blood and enrichment blood culture DNA samples, obtained from 82 individuals previously tested in our laboratory [1,36,80,81,82,83,84], were used to assess the relative sensitivity and specificity of four amplification modalities (18S rRNA qPCR, 18S rRNA dPCR, *Babesia* ITS1 qPCR, and *Babesia* ITS2 qPCR). These samples were analyzed to determine the frequency of positive DNA amplification and to assess whether the sequence quality (available for qPCR testing only) was adequate to obtain a readable DNA sequence.

From the 226 human DNA blood and enrichment blood culture samples tested, 19 (8.4%) were positive by qPCR targeting Piroplasmida 18S rRNA; 70 (31%) were positive by dPCR targeting Piroplasmida 18S rRNA; 29 (12.8%) were positive by qPCR targeting *Babesia* ITS1; and 40 (17.7%) were positive by qPCR targeting *Babesia* ITS2 (Table 1).

DNA sequence analysis (using Clustal W multi-sequence alignment; AlignX, Vector NTI Advance 10.3.0 from Invitrogen) was not successful for any of the 18S rRNA qPCR-positive samples, precluding species identification, whereas *Babesia* DNA was successfully sequenced from 26 of 29 *Babesia* ITS1 qPCR and 20 of 40 *Babesia* ITS2 qPCR samples, respectively.

In most instances, *Babesia* ITS1 qPCR rendered higher-quality DNA sequences (using both forward and reverse primers) compared to the *Babesia* ITS2 qPCR for the same DNA extraction. Based upon these comparative ITS1 and ITS2 results, the *Babesia* ITS1 region was selected for the detection of Piroplasmida at the genus level and for the design of species-specific primers and probes for the detection of *B. divergens*, *B. duncani*, *B. microti*, and *B. odocoilei*, which are the most frequently documented human *Babesia* spp. pathogens in the USA.

### 3.2. Development of B. divergens, B. duncani, B. microti, and B. odocoilei ITS1 Species-Specific Primers and Probes

**Figure 2:**
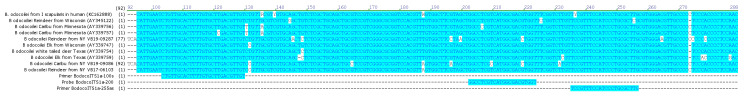
Alignments of *B odocoilei* ITS1 sequence region.

**Figure 3:**
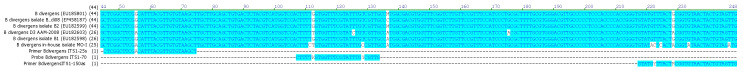
Alignments of *B. divergens* ITS1 region.

**Figure 4:**
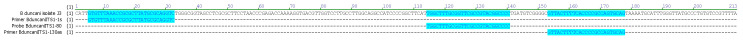
Alignments of *B duncani* ITS1 region. No *B duncani* ITS1 region available from GenBank database.

**Figure 5:**
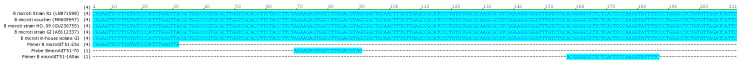
Alignments of *B microti* ITS1 region.

After DNA sequence analysis of PCR amplicons obtained from *B. odocoilei*-positive animal blood specimens (Figure 2), from *B. divergens*-like isolate MO-I (Figure 3), from *B. duncani* isolate J3 (Figure 4), and from *B. microti* isolate GI (Figure 5), the following species-specific primers and probes for amplification of the ITS1 region were designed and subsequently tested for amplification specificity:

*Babesia divergens* ITS1 target region (225 bp):
  Primer BdivergensITS1-25s: 5′ CTCGGCTTCGACATTTACGTTGTGTAAGCT 3′
  Primer BdivergensITS1-150as: 5′ CAACTACAGTAGTTACACCGYAGTAARCATAC 3′
  Probe BdivergensITS1-70: 5′ HEX CTTTTKGTGGTTTCGTATTTGYCGTTG-BHQ2 3′
*Babesia duncani* ITS1 target region (170 bp):
  Primer BduncaniTS1-1s: 5′ GTGTTTAAACCGCGCTTATGCGCAGGTC 3′
  Primer BduncaniTS1-130as: 5′ CTGCACTGGCGGGGTGAAAAGTAAC 3′
  Probe BduncaniTS1-80: 5′ Cy5-TGGCTTTGCGGTTCGCCGTACGGCCCC-BHQ3 3′
*Babesia microti* ITS1 target region (185 bp):
  Primer BmicrotiITS1-25s: 5′ TATCAGAGTTCTTTGTATCCCATTTGGGTTA 3′
  Primer BmicrotiITS1-160as: 5′ GAAAATACCTTGGGAGTGAGAACGCCCCGT 3′
  Probe BmicrotiITS1-70: 5′ CalFluoRed590-AGAAGAGTGGCCTTGGACGTAG-BHQ2 3′
*Babesia odocoilei* ITS1 target region (150 bp):
  Primer BodocoITS1a-100s: 5′ CTGTTGCACTTTTGTGCTTGACGTTGT 3′
  Primer BodocoITS1a-255as: 5′ CAAGCGCAGGGATGGAAACGGA 3′
  Probe BodocoITS1a-200probe: 5′ FAM-GGCCTCGTCATGGCGACGTGGT-BHQ1 3′

For both TaqMan probes and SYBR Green-based qPCR, species-specific ITS1 amplification was detectable at levels of 10^−2^ (for *B. microti* and *B. odocoilei*) and 10^−1^ (for *B. divergens* and *B. duncani*) copies per microliter for each of the four targeted *Babesia* spp. Amplicons of the lowest DNA concentration generated good-quality chromatograms with sequences that matched the targeted *Babesia* sp. Melting curve analyses for each species by SYBR Green qPCR generated peaks at 89.5 °C to 90 °C for *B. odocoilei*, 86.5 °C for *B. divergens*, 89 °C for *B. duncani*, and 88.5 °C for *B. microti*.

### 3.3. Assessment of Species-Specific Amplification for Cross-Amplification with Non-Target Babesia *spp*.

There was no cross-amplification using either TaqMan probes or SYBRGreen-based qPCR assays, when *B. divergens*, *B. duncani*, *B. microti*, and *B. odocoilei* species-specific primers and probes were tested against DNA extracted from the blood of animals infected with *B. vulpes, B. odocoilei*, *B. canis*, *B. vogeli*, *Babesia* sp. Coco, *B. gibsoni*, *B. lengau*, *B. felis*, pr *B. conradae* or when tested against *B. microti*, *B. divergens*, *B. odocoilei*, or *B. duncani* plasmid-based DNA (Table S3).

### 3.4. Detection of Babesia *spp*. Infection in Human Samples

Of the 82 human research subjects tested during this assay validation study, 22 (26.8%) were infected with one or more *Babesia* species. Amplicon DNA sequence analysis of qPCR products targeting the *Babesia* ITS1 and/or *Babesia* ITS2 regions identified single infection with *Babesia divergens* in seven (8.5%) individuals, *B. odocoilei* in seven (8.5%) individuals, or *B. microti* in two (2.4%) individuals (Table S3). As a result of analyzing different samples (different time points within 7-day blood collections or blood vs. 7-, 14-, or 21-day enrichment culture samples) from the same individual, *Babesia* spp. co-infection was detected in six (7.3%) individuals, as well as two (2.4%) individuals infected with *B. divergens* and *B. microti*, three (3.7%) individuals infected with *B. divergens* and *B. odocoilei*, and one (1.2%) individual infected with *B. microti* and *B. odocoilei* (Table 2).

**Figure 6:**
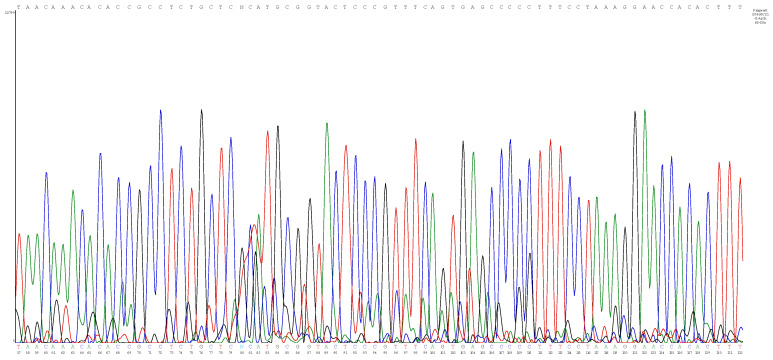
Overlapping sequencing chromatograms (double peaks) obtained from the enrichment blood culture sample 20468C21 amplicon from a human patient obtained by *Babesia* ITS1 amplification, indicating potential alternative allele sequences due to co-infection, which was confirmed as *B. divergens* and *B. odocoilei* using species-specific ITS1 probes.

For many of these individuals, the same *Babesia* species was detected at different blood sampling time points. Sequence analysis of some qPCR products obtained from people where co-infection was documented indicated the presence of mixed sequencing chromatograms (usually double peaks with one chromatogram as a low signal, as shown in Figure 6), indicating the potential for alternative allele sequences. To assess if these amplicons indicated co-infection with more than one *Babesia* sp. (beyond the species previously identified by qPCR and DNA sequencing), a single-plex species-specific real-time PCR targeting the *Babesia* ITS1 region was developed. Using the qPCR single-plex assay targeting ITS-1 of the four selected zoonotic *Babesia* sp., co-infection with more than one *Babesia* sp. was confirmed by DNA sequencing, as these patient samples contained alternative allele sequences. For other individuals, the single-plex assay amplified sequences of the same species (i.e., *B. divergens* or *B. odocoilei*) previously identified by the *Babesia* ITS1 or ITS2 amplification. *Babesia odocoilei* was the “secondary” co-infecting species identified by species-specific ITS1 region amplification in all eight individuals, with amplifications of very high threshold cycle (Ct) values (>37 to over 40 total cycles), indicating a very low-level parasitemia. Co-infection with *B. divergens* and *B. odocoilei* was most frequently detected (seven of eight individuals), as illustrated by the patient depicted in Figure 6 for enrichment culture sample 20468C21. Another individual was co-infected with *B. microti* and *B. odocoilei*.

*Babesia* sequences of amplicons (ITS1 and ITS2) obtained from these individuals have been deposited in the GenBank database under the following accession numbers: *B. odocoilei*: PP693407, PP693408, PP693409, PQ452565, PQ452566, PQ452567, PQ452568, PQ452569, PQ452570, PP550653, PP550654, PP550655, PP550656, PP550657, PP550658, PP550659, PP550660, PP550661, PP592351, PP550644, PP550645, PP550646, PP550647, PP550648, PP550649, PP550650, PP550651, PP550652, PQ452561, PQ452562, PQ452563, and PQ452564; *B. divergens*: PQ404846, PQ404847, PQ404848, PP693420, PP693421, PP693422, PP693423, PP693424, PP693425, PP693426, PQ452546, PQ452547, PQ452548, PQ394580, PQ459266, PQ459267, PQ459268, PQ459269, PQ459270, PQ459271, PQ459272, PQ459273, PQ459274, and PQ459275; *B. microti*: PQ459008, PQ459009, PQ459010, PQ459011, PQ459012, PP693410, PP693411, PP693412, PP693413, PP693414, PP693415, PQ452571, PQ452572, PP693416, PP693417, PP693418, PP693419, PQ452549, PQ452550, PQ452551, PQ452552, PQ452553, PQ452554, PQ452555, PQ452556, PQ452557, PQ452558, PQ452559, PQ452560, PQ459293, PQ459262, PQ459263, PQ459264, and PQ459265.

*Babesia duncani* DNA was not amplified as a single or co-infection in any of the 226 human patient samples analyzed in this study.

## 4. Discussion

Using blood specimens from animals naturally infected with piroplasmids, experimentally infected animals, and human clinical samples derived from research studies, specific qPCR assays were designed and validated for amplification of the *Babesia* intergenic region (ITS1) at the genus, species-specific, and strain levels, specifically targeting *B. odocoilei*, *B. duncani*, *B. divergens*, and *B. microti*. In previously published human babesiosis case reports confirmed by DNA sequencing, the diagnosis was most often confirmed by targeting the highly conserved 18S rRNA gene, which is a good marker for phylogenetic inferences, especially at the taxon level. However, failure to obtain a confirmatory DNA sequence from positive 18S rRNA gene amplicons occurs frequently, which negatively impacts the possibility of achieving a definitive species identification. Failure to successfully sequence an 18S rRNA qPCR amplicon is most often due to low DNA quantity, a finding consistent with testing results reported in this study, as most samples rendered very high qPCR threshold cycle (Ct) values (>37 over 40 total cycles). In addition, non-specific amplification or co-infection with two *Babesia* species can also contribute to sequencing failure.

The *Babesia* spp. intergenic spacer regions have greater evolutionary variation when compared to the conserved 18S rRNA gene. This genetic variation is associated with the facilitation of more rapid genetic change, making the ITS regions useful for defining species and microbial populations at a more refined specific species or strain level [12,13,38]. The ITS region has proven useful for diagnosing closely related species of *Cystoisospora* [12], as well as being used as a PCR target for dinoflagellates [15], fungi [14,39], Thalassiosirales [38], and nematodes [40,41]. Previously, Wilson et al. (2005) [65] developed qPCR and ddPCR protocols based on the ITS1 region of *B. microti* and *B. duncani*.

The primers and probes developed in this study proved to be specific for the four *Babesia* spp. investigated, with no cross-amplification with any other tested Piroplasmids species. The ITS1 TaqMan probe and SYBRGreen-based assays proved to be sensitive, being able to detect a minimum of 10^−1^ copies per μL. The use of more sensitive and specific assays, primarily aimed at detecting zoonotic *Babesia* species, favors a more accurate, species-specific diagnosis, which could impact treatment decisions and preventive strategies. In addition, the use of sensitive and specific species primers or probes facilitates the detection of *Babesia* spp. co-infections, as reported in this study, which may go undetected when targeting the more evolutionarily conserved 18S rRNA gene. In the initial studies carried out by our research group, DNA extracted from the blood of cervids was positive by conventional 18S rRNA PCR, and DNA sequencing confirmed infection with *Theileria cervi*. These same cervid blood samples were also positive using the *B. odocoilei*-specific ITS1 qPCR assay, with species confirmation determined by DNA sequencing. Thus, co-infection was confirmed in reservoir hosts, results that would not have been obtained by solely targeting the 18S rRNA gene (Figures S1–S3).

Currently, babesiosis is increasingly being diagnosed in humans, often in association with a diagnosis of Lyme borreliosis, transmitted by *Ixodes* ticks [2,5,36,85]. As reviewed, the initial recognition and molecular characterization of different *Babesia* species infecting humans is also increasingly reported in the worldwide medical literature [43]. The most recent descriptions and sequencing confirmation were of *B. odocoilei* detected in symptomatic humans from Canada and the USA [1,42]. With the advent of molecular assays, a more accurate diagnosis of the specific *Babesia* species infecting animals and human patients has become possible, confirming substantially greater diversity of species circulating in and among animals with the possibility of spillover to humans. In the past, most human *Babesia* studies were based on direct microscopic visualization or serological assays, so most cases of babesiosis were mainly attributed to *B. microti* [28,44,45,47,49,50,51,52,86,87]. However, the possibility of serological cross-reactivity among *Babesia* spp. cannot be ruled out, as well as co-infection with more than one *Babesia* species that would not likely be diagnosed clinically. More specific diagnostic tests, such as those targeting the ITS1 *Babesia* genus and species-specific assays developed in this study, will favor diagnosis at the species level and will facilitate documentation of *Babesia* and Piroplasmida co-infections diagnostically and in epidemiological studies. The bite of an infected tick is described as the main route of *Babesia* spp. transmission, followed by blood transfusion and tissue transplants [27,53,88]. However, other potential transmission routes should not be ruled out. For example, there is evidence of *B. gibsoni* being transmitted through fighting among American Staffordshire (Pitbull) terriers [69], indicating the possibility of direct transmission between animals and humans.

In addition to the development of targeted *Babesia* spp. qPCR assays through this research, we generated and submitted to GenBank complete sequences of the ITS-1 and ITS-2 regions of different Piroplasmida species (*B. vulpes*, *B. canis*, *B. vogeli*, *Babesia* sp. Coco, *B. gibsoni*, *B. lengau*, *B. felis*, *B. odocoilei*, *B. divergens*, *B. capreoli*, *B. negevi*, *T. bicornis* and *C. felis*), including many ITS regions that had never been previously sequenced. Access to these sequences will contribute to future studies aimed at developing and validating molecular assays targeting these regions.

Based on the use of PCR amplification and sequencing of the *Babesia* ITS1 region (both at genus and species levels), we were able to identify not just *B. odocoilei*, *B. divergens*, and *B. microti* in studied participants, most of whom reported chronic and often non-specific symptoms, but also co-infections with two *Babesia* species in a small subgroup of individuals. Interestingly, *B. duncani* DNA was not detected as either single or co-infection in any of the 226 human samples analyzed in this study. As the purpose of this study was to describe improvements in methodology for the detection and molecular characterization of *Babesia* spp., medical details regarding the 82 study participants will be reported in future publications, as these assays were used to generate *B. odocoilei* DNA sequences for a recent publication [1].

## 5. Conclusions

Using the intergenic spacer regions between the 18S rRNA and the 5.8S rRNA (known as ITS1) and between the 5.8S rRNA and the 28S rRNA genes (known as ITS2), we designed a set of highly sensitive molecular primers and probes that facilitate the amplification, detection, and characterization of *Babesia* spp. that infect humans and animals. The primers and probes designed for species-specific detection (here targeting *B. divergens*, *B. duncani*, *B. microti*, and *B. odocoilei*) will facilitate the diagnosis of single and co-infections with *Babesia* spp. in animals and human patients.

## Figures and Tables

**Table 1:** Prevalence of *Babesia* species as positive DNA amplification using qPCR and dPCR aiming at 18S rRNA gene and qPCR aiming at the 18S rRNA-5.8S rRNA intergenic (ITS1) region, in 226 human DNA blood and enrichment blood culture samples.

	Piroplasmida 18S rRNA		*Babesia* ITS1
	qPCR	dPCR	qPCR
Negatives	207	156	197
Positives	19	70	29

**Table 2:** *Babesia* species detected in 22 of 82 individuals tested as a component of this study.

Species Detected and Sequenced	Individuals
*B. divergens*	7
*B. microti*	2
*B. odocoilei*	7
*B. divergens* and *B. microti*	2
*B. divergens* and *B. odocoilei*	3
*B. microti* and *B. odocoilei*	1

## Data Availability

Data on reference human results are unavailable due to privacy and ethical restrictions (IRB#s 4925-03 and 164-08-05). All other data are available upon request.

## Supplementary Materials

The following supporting information can be downloaded at: https://www.mdpi.com/article/10.3390/pathogens13121094/s1, Figure S1: Electropherogram of an 18S rRNA gene sequence showing the overlapping peaks; Figure S2: Part of the 18S rRNA alignment of *T. cervi*, *B. odocoilei* and deer sequences showing great divergence between *T. cervi* and *B. odocoilei*; Figure S3: Alignment of ITS-1 sequences; Table S1: *Babesia* species currently identified by DNA sequence infecting people worldwide; Table S2: Piroplasmida species, animal source, case identification and GenBank Accession numbers generated in this study for the intergenic transcribed spacer regions between 18S rRNA and 5.8S rRNA (ITS1) and 5.8S rRNA and 28S rRNA (ITS2). Note: N/A: not amplified; Table S3: Assessment of *Babesia* species specificity and cross-amplification with other *Babesia* species for the species-specific ITS1 assay. Note: original refers to DNA extracted from blood from pre-characterized animal clinical samples.

## References

[1] Saito-Ito A., Tsuji M., Wei Q., He S., Matsui T., Kohsaki M., Arai S., Kamiyama T., Hioki K., Ishihara C. (2000). Transfusion-acquired, autochthonous human babesiosis in Japan: Isolation of *Babesia microti*-like parasites with hu-RBC-SCID mice. J. Clin. Microbiol..

[2] Beattie J.F., Michelson M.L., Holman P.J. (2002). Acute babesiosis caused by *Babesia divergens* in a resident of Kentucky. N. Engl. J. Med..

[3] Shock B.C., Moncayo A., Cohen S., Mitchell E.A., Williamson P.C., Lopez G., Garrison L.E., Yabsley M.J. (2014). Diversity of piroplasms detected in blood-fed and questing ticks from several states in the United States. Ticks Tick-Borne Dis..

[4] França R.T., Da Silva A.S., Loretti A.P., Mazzanti C.M., Lopes S.T. (2014). Canine rangeliosis due to *Rangelia vitalii*: From first report in Brazil in 1910 to current day—A review. Ticks Tick-Borne Dis..

[5] Calvopiña M., Montesdeoca-Andrade M., Bastidas-Caldes C., Enriquez S., Rodríguez-Hidalgo R., Aguilar-Rodriguez D., Cooper P. (2023). Case report: First report on human infection by tick-borne Babesia bigemina in the Amazon region of Ecuador. Front. Public Health.

[6] Abdoli A., Olfatifar M., Badri M., Zaki L., Bijani B., Pirestani M., Hatam-Nahavandi K., Eslahi A.V., Karanis P. (2024). A global systematic review and meta-analysis on the babesiosis in dogs with special reference to *Babesia canis*. Vet. Med. Sci..

[7] Hussain S., Hussain A., Aziz M.U., Song B., Zeb J., George D., Li J., Sparagano O. (2021). A Review of Zoonotic Babesiosis as an Emerging Public Health Threat in Asia. Pathogens.

[8] Panti-May J.A., Rodríguez-Vivas R.I. (2020). Canine babesiosis: A literature review of prevalence, distribution, and diagnosis in Latin America and the Caribbean. Vet. Parasitol. Reg. Stud. Rep..

[9] Saleh A.M., Adam S.M., Abdel-Motagaly A.M., Ibrahim A., Morsy T.A. (2015). Cot. J. Egypt. Soc. Parasitol..

[10] Solano-Gallego L., Sainz A., Roura X., Estrada-Pena A., Miro G. (2016). A review of canine babesiosis: The European perspective. Parasites Vectors.

[11] Schnittger L., Rodriguez A.E., Florin-Christensen M., Morrison D.A. (2012). Babesia: A world emerging. Infect. Genet. Evol..

[12] Jia N., Zheng Y.C., Jiang J.F., Jiang R.R., Jiang B.G., Wei R., Liu H.B., Huo Q.B., Sun Y., Chu Y.L. (2018). Human Babesiosis Caused by a *Babesia crassa*-like Pathogen: A Case Series. Clin. Infect. Dis..

[13] Doderer-Lang C., Filisetti D., Badin J., Delale C., Clavier V., Brunet J., Gommenginger C., Abou-Bacar A., Pfaff A.W. (2022). *Babesia crassa*-like Human Infection Indicating Need for Adapted PCR Diagnosis of Babesiosis, France. Emerg. Infect. Dis..

[14] Jahfari S., Hofhuis A., Fonville M., van der Giessen J., van Pelt W., Sprong H. (2016). Molecular Detection of Tick-Borne Pathogens in Humans with Tick Bites and Erythema Migrans, in the Netherlands. PLoS Negl. Trop. Dis..

[15] Gonzalez L.M., Castro E., Lobo C.A., Richart A., Ramiro R., Gonzalez-Camacho F., Luque D., Velasco A.C., Montero E. (2015). First report of *Babesia divergens* infection in an HIV patient. Int. J. Infect. Dis..

[16] Livengood J., Hutchinson M.L., Thirumalapura N., Tewari D. (2020). Detection of *Babesia*, *Borrelia*, *Anaplasma*, and *Rickettsia* spp. in Adult Black-Legged Ticks (*Ixodes scapularis*) from Pennsylvania, United States, with a Luminex Multiplex Bead Assay. Vector-Borne Zoonotic Dis..

[17] Milnes E.L., Thornton G., Leveille A.N., Delnatte P., Barta J.R., Smith D.A., Nemeth N. (2019). *Babesia odocoilei* and zoonotic pathogens identified from *Ixodes scapularis* ticks in southern Ontario, Canada. Ticks Tick-Borne Dis..

[18] Scott J.D., Clark K.L., Durden L.A. (2019). Presence of *Babesia odocoilei* and *Borrelia burgdorferi* Sensu Stricto in a Tick and Dual Parasitism of *Amblyomma inornatum* and *Ixodes scapularis* on a Bird in Canada. Healthcare.

[19] Scott J.D., Pascoe E.L., Sajid M.S., Foley J.E. (2020). Detection of *Babesia odocoilei* in *Ixodes scapularis* Ticks Collected from Songbirds in Ontario and Quebec, Canada. Pathogens.

[20] Scott J.D., Pascoe E.L., Sajid M.S., Foley J.E. (2021). Detection of *Babesia odocoilei* in *Ixodes scapularis* Ticks Collected in Southern Ontario, Canada. Pathogens.

[21] Scott J.D., Pesapane R.R. (2021). Detection of *Anaplasma phagocytophilum*, *Babesia odocoilei*, *Babesia* sp., *Borrelia burgdorferi* Sensu Lato, and *Hepatozoon canis* in *Ixodes scapularis* Ticks Collected in Eastern Canada. Pathogens.

[22] Steiner F.E., Pinger R.R., Vann C.N., Abley M.J., Sullivan B., Grindle N., Clay K., Fuqua C. (2006). Detection of *Anaplasma phagocytophilum* and *Babesia odocoilei* DNA in *Ixodes scapularis* (Acari: Ixodidae) collected in Indiana. J. Med. Entomol..

[23] Waldrup K.A., Kocan A.A., Barker R.W., Wagner G.G. (1990). Transmission of *Babesia odocoilei* in white-tailed deer (*Odocoileus virginianus*) by *Ixodes scapularis* (Acari: Ixodidae). J. Wildl. Dis..

[24] Zembsch T.E., Bron G.M., Paskewitz S.M. (2021). Evidence for Vertical Transmission of *Babesia odocoilei* (Piroplasmida: Babesiidae) in *Ixodes scapularis* (Acari: Ixodidae). J. Med. Entomol..

[25] Gandy S., Medlock J., Cull B., Smith R., Gibney Z., Sewgobind S., Parekh I., Harding S., Johnson N., Hansford K. (2024). Detection of Babesia species in questing *Ixodes ricinus* ticks in England and Wales. Ticks Tick-Borne Dis..

[26] Gray A., Capewell P., Zadoks R., Taggart M.A., French A.S., Katzer F., Shiels B.R., Weir W. (2021). Wild deer in the United Kingdom are a potential reservoir for the livestock parasite *Babesia divergens*. Curr. Res. Parasitol. Vector-Borne Dis..

[27] Jain K., Tagliafierro T., Marques A., Sanchez-Vicente S., Gokden A., Fallon B., Mishra N., Briese T., Kapoor V., Sameroff S. (2021). Development of a capture sequencing assay for enhanced detection and genotyping of tick-borne pathogens. Sci. Rep..

[28] Gabrielli S., Totino V., Macchioni F., Zuñiga F., Rojas P., Lara Y., Roselli M., Bartoloni A., Cancrini G. (2016). Human Babesiosis, Bolivia, 2013. Emerg. Infect. Dis..

[29] Brennan M.B., Herwaldt B.L., Kazmierczak J.J., Weiss J.W., Klein C.L., Leith C.P., He R., Oberley M.J., Tonnetti L., Wilkins P.P. (2016). Transmission of *Babesia microti* Parasites by Solid Organ Transplantation. Emerg. Infect. Dis..

[30] Vannier E., Krause P.J. (2012). Human babesiosis. N. Engl. J. Med..

[31] Corduneanu A., Ursache T.D., Taulescu M., Sevastre B., Modrý D., Mihalca A.D. (2020). Detection of DNA of *Babesia canis* in tissues of laboratory rodents following oral inoculation with infected ticks. Parasites Vectors.

[32] Joseph J.T., Purtill K., Wong S.J., Munoz J., Teal A., Madison-Antenucci S., Horowitz H.W., Aguero-Rosenfeld M.E., Moore J.M., Abramowsky C. (2012). Vertical transmission of *Babesia microti*, United States. Emerg. Infect. Dis..

[33] Malagon F., Tapia J.L. (1994). Experimental transmission of *Babesia microti* infection by the oral route. Parasitol. Res..

[34] Mierzejewska E.J., Welc-Falęciak R., Bednarska M., Rodo A., Bajer A. (2014). The first evidence for vertical transmission of *Babesia canis* in a litter of Central Asian Shepherd dogs. Ann. Agric. Environ. Med..

[35] Tołkacz K., Bednarska M., Alsarraf M., Dwużnik D., Grzybek M., Welc-Falęciak R., Behnke J.M., Bajer A. (2017). Prevalence, genetic identity and vertical transmission of *Babesia microti* in three naturally infected species of vole, *Microtus* spp. (*Cricetidae*). Parasites Vectors.

[36] Espinosa-Muñoz D.Y., López-López L., Ríos-Osorio L.A., Gutiérrez L.A. (2022). Detection of Babesia and the associated factors in cattle and humans from Magdalena Medio region, Colombia. Comp. Immunol. Microbiol. Infect. Dis..

[37] Haapasalo K., Suomalainen P., Sukura A., Siikamäki H., Jokiranta T.S. (2010). Fatal babesiosis in man, Finland, 2004. Emerg. Infect. Dis..

[38] Strasek-Smrdel K., Korva M., Pal E., Rajter M., Skvarc M., Avsic-Zupanc T. (2020). Case of *Babesia crassa*-like Infection, Slovenia, 2014. Emerg. Infect. Dis..

[39] Kukina I.V., Guzeeva T.M., Zelya O.P., Ganushkina L.A. (2018). Fatal human babesiosis caused by *Babesia divergens* in an asplenic host. IDCases.

[40] Martinot M., Zadeh M.M., Hansmann Y., Grawey I., Christmann D., Aguillon S., Jouglin M., Chauvin A., De Briel D. (2011). Babesiosis in immunocompetent patients, Europe. Emerg. Infect. Dis..

[41] Qi C., Zhou D., Liu J., Cheng Z., Zhang L., Wang L., Wang Z., Yang D., Wang S., Chai T. (2011). Detection of *Babesia divergens* using molecular methods in anemic patients in Shandong Province, China. Parasitol. Res..

[42] Centeno-Lima S., do Rosario V., Parreira R., Maia A.J., Freudenthal A.M., Nijhof A.M., Jongejan F. (2003). A fatal case of human babesiosis in Portugal: Molecular and phylogenetic analysis. Trop. Med. Int. Health.

[43] Wang J., Zhang S., Yang J., Liu J., Zhang D., Li Y., Luo J., Guan G., Yin H. (2019). *Babesia divergens* in human in Gansu province, China. Emerg. Microbes Infect..

[44] Herwaldt B.L., de Bruyn G., Pieniazek N.J., Homer M., Lofy K.H., Slemenda S.B., Fritsche T.R., Persing D.H., Limaye A.P. (2004). *Babesia divergens*-like infection, Washington State. Emerg. Infect. Dis..

[45] Bloch E.M., Herwaldt B.L., Leiby D.A., Shaieb A., Herron R.M., Chervenak M., Reed W., Hunter R., Ryals R., Hagar W. (2012). The third described case of transfusion-transmitted *Babesia duncani*. Transfusion.

[46] Conrad P.A., Kjemtrup A.M., Carreno R.A., Thomford J., Wainwright K., Eberhard M., Quick R., Telford S.R., Herwaldt B.L. (2006). Description of *Babesia duncani* n.sp. (Apicomplexa: Babesiidae) from humans and its differentiation from other piroplasms. Int. J. Parasitol..

[47] Arsuaga M., Gonzalez L.M., Lobo C.A., de la Calle F., Bautista J.M., Azcárate I.G., Puente S., Montero E. (2016). First Report of *Babesia microti*-Caused Babesiosis in Spain. Vector-Borne Zoonotic Dis..

[48] Gilmore R.D., Carpio A.M., Kosoy M.Y., Gage K.L. (2003). Molecular characterization of the sucB gene encoding the immunogenic dihydrolipoamide succinyltransferase protein of *Bartonella vinsonii* subsp. *berkhoffii* and *Bartonella quintana*. Infect. Immun..

[49] Hildebrandt A., Hunfeld K.P., Baier M., Krumbholz A., Sachse S., Lorenzen T., Kiehntopf M., Fricke H.J., Straube E. (2007). First confirmed autochthonous case of human *Babesia microti* infection in Europe. Eur. J. Clin. Microbiol. Infect. Dis..

[50] Holler J.G., Röser D., Nielsen H.V., Eickhardt S., Chen M., Lester A., Bang D., Frandsen C., David K.P. (2013). A case of human babesiosis in Denmark. Travel Med. Infect. Dis..

[51] Moniuszko-Malinowska A., Swiecicka I., Dunaj J., Zajkowska J., Czupryna P., Zambrowski G., Chmielewska-Badora J., Żukiewicz-Sobczak W., Swierzbinska R., Rutkowski K. (2016). Infection with *Babesia microti* in humans with non-specific symptoms in North East Poland. Infect. Dis..

[52] Jabłońska J., Żarnowska-Prymek H., Stańczak J., Kozłowska J., Wiercińska-Drapało A. (2016). Symptomatic co-infection with *Babesia microti* and *Borrelia burgdorferi* in patient after international exposure; A challenging case in Poland. Ann. Agric. Environ. Med..

[53] Kim H.J., Kim M.J., Shin H.I., Ju J.W., Lee H.I. (2023). Imported human babesiosis in the Republic of Korea, 2019: Two case reports. Parasites Hosts Dis..

[54] Kim J.Y., Cho S.H., Joo H.N., Tsuji M., Cho S.R., Park I.J., Chung G.T., Ju J.W., Cheun H.I., Lee H.W. (2007). First case of human babesiosis in Korea: Detection and characterization of a novel type of *Babesia* sp. (KO1) similar to ovine babesia. J. Clin. Microbiol..

[55] Peniche-Lara G., Balmaceda L., Perez-Osorio C., Munoz-Zanzi C. (2018). Human Babesiosis, Yucatán State, Mexico, 2015. Emerg. Infect. Dis..

[56] Sayama Y., Zamoto-Niikura A., Matsumoto C., Saijo M., Ishihara C., Matsubayashi K., Nagai T., Satake M. (2018). Analysis of antigen-antibody cross-reactivity among lineages and sublineages of *Babesia microti* parasites using human babesiosis specimens. Transfusion.

[57] Senanayake S.N., Paparini A., Latimer M., Andriolo K., Dasilva A.J., Wilson H., Xayavong M.V., Collignon P.J., Jeans P., Irwin P.J. (2012). First report of human babesiosis in Australia. Med. J. Aust..

[58] Stahl P., Poinsignon Y., Pouedras P., Ciubotaru V., Berry L., Emu B., Krause P.J., Ben Mamoun C., Cornillot E. (2018). Case report of the patient source of the *Babesia microti* R1 reference strain and implications for travelers. J. Travel Med..

[59] Welc-Falęciak R., Pawełczyk A., Radkowski M., Pancewicz S.A., Zajkowska J., Siński E. (2015). First report of two asymptomatic cases of human infection with *Babesia microti* (Franca, 1910) in Poland. Ann. Agric. Environ. Med..

[60] Zhou X., Li S.G., Wang J.Z., Huang J.L., Zhou H.J., Chen J.H., Zhou X.N. (2014). Emergence of human babesiosis along the border of China with Myanmar: Detection by PCR and confirmation by sequencing. Emerg. Microbes Infect..

[61] Zhou X., Li S.G., Chen S.B., Wang J.Z., Xu B., Zhou H.J., Ge H.X., Chen J.H., Hu W. (2013). Co-infections with *Babesia microti* and Plasmodium parasites along the China-Myanmar border. Infect. Dis. Poverty.

[62] Huang L., Sun Y., Huo D.D., Xu M., Xia L.Y., Yang N., Hong W., Nie W.M., Liao R.H., Zhang M.Z. (2023). Successful treatment with doxycycline monotherapy for human infection with *Babesia venatorum* (Babesiidae, Sporozoa) in China: A case report and proposal for a clinical regimen. Infect. Dis. Poverty.

[63] Jiang J.F., Zheng Y.C., Jiang R.R., Li H., Huo Q.B., Jiang B.G., Sun Y., Jia N., Wang Y.W., Ma L. (2015). Epidemiological, clinical, and laboratory characteristics of 48 cases of “*Babesia venatorum*” infection in China: A descriptive study. Lancet Infect. Dis..

[64] Sun Y., Li S.G., Jiang J.F., Wang X., Zhang Y., Wang H., Cao W.C. (2014). *Babesia venatorum* Infection in Child, China. Emerg. Infect. Dis..

[65] Hong S.H., Kim S.Y., Song B.G., Roh J.Y., Cho C.R., Kim C.N., Um T.H., Kwak Y.G., Cho S.H., Lee S.E. (2019). Detection and characterization of an emerging type of *Babesia* sp. similar to *Babesia motasi* for the first case of human babesiosis and ticks in Korea. Emerg. Microbes Infect..

[66] Häselbarth K., Tenter A.M., Brade V., Krieger G., Hunfeld K.P. (2007). First case of human babesiosis in Germany—Clinical presentation and molecular characterisation of the pathogen. Int. J. Med. Microbiol..

[67] Bonsergent C., de Carné M.C., de la Cotte N., Moussel F., Perronne V., Malandrin L. (2021). The New Human *Babesia* sp. FR1 Is a European Member of the *Babesia* sp. MO1 Clade. Pathogens.

[68] Man S.Q., Qiao K., Cui J., Feng M., Fu Y.F., Cheng X.J. (2016). A case of human infection with a novel *Babesia* species in China. Infect. Dis. Poverty.

[69] Herwaldt B.L., Cacciò S., Gherlinzoni F., Aspöck H., Slemenda S.B., Piccaluga P., Martinelli G., Edelhofer R., Hollenstein U., Poletti G. (2003). Molecular characterization of a non-*Babesia divergens* organism causing zoonotic babesiosis in Europe. Emerg. Infect. Dis..

[70] Yang Y., Christie J., Köster L., Du A., Yao C. (2021). Emerging Human Babesiosis with “Ground Zero” in North America. Microorganisms.

[71] Waked R., Krause P.J. (2022). Human Babesiosis. Infect. Dis. Clin. N. Am..

[72] Locke S., O’Bryan J., Zubair A.S., Rethana M., Moffarah A.S., Krause P.J., Farhadian S.F. (2023). Neurologic Complications of Babesiosis, United States, 2011–2021. Emerg. Infect. Dis..

[73] Baneth G., Kenny M.J., Tasker S., Anug Y., Shkap V., Levy A., Shaw S.E. (2004). Infection with a proposed new subspecies of *Babesia canis*, *Babesia canis* subsp. *presentii*, in domestic cats. J. Clin. Microbiol..

[74] Wilson M., Glaser K.C., Adams-Fish D., Boley M., Mayda M., Molestina R.E. (2015). Development of droplet digital PCR for the detection of *Babesia microti* and *Babesia duncani*. Exp. Parasitol..

[75] Calchi A.C., Yogui D.R., Alves M.H., Desbiez A.L.J., Kluyber D., Vultão J.G., Arantes P.V.C., de Santi M., Werther K., Teixeira M.M.G. (2023). Molecular detection of piroplasmids in mammals from the Superorder Xenarthra in Brazil. Parasitol. Res..

[76] Bloch E.M., Kumar S., Krause P.J. (2019). Persistence of *Babesia microti* Infection in Humans. Pathogens.

[77] Krause P.J. (2019). Human babesiosis. Int. J. Parasitol..

[78] Radcliffe C., Krause P.J., Grant M. (2019). Repeat exchange transfusion for treatment of severe babesiosis. Transfus. Apher. Sci..

[79] Maggi R., Breitschwerdt E.B., Qurollo B., Miller J.C. (2021). Development of a Multiplex Droplet Digital PCR Assay for the Detection of *Babesia*, *Bartonella*, and *Borrelia* Species. Pathogens.

[80] Lashnits E., Maggi R., Jarskog F., Bradley J., Breitschwerdt E., Frohlich F. (2021). Schizophrenia and *Bartonella* spp. Infection: A Pilot Case-Control Study. Vector-Borne Zoonotic Dis..

[81] Portillo A., Maggi R., Oteo J.A., Bradley J., García-Álvarez L., San-Martín M., Roura X., Breitschwerdt E. (2020). *Bartonella* spp. Prevalence (Serology, Culture, and PCR) in Sanitary Workers in La Rioja Spain. Pathogens.

[82] Breitschwerdt E.B., Bradley J.M., Maggi R.G., Lashnits E., Reicherter P. (2020). Bartonella Associated Cutaneous Lesions (BACL) in People with Neuropsychiatric Symptoms. Pathogens.

[83] Oteo J.A., Maggi R., Portillo A., Bradley J., García-Álvarez L., San-Martín M., Roura X., Breitschwerdt E. (2017). Prevalence of *Bartonella* spp. by culture, PCR and serology, in veterinary personnel from Spain. Parasites Vectors.

[84] Lantos P.M., Maggi R.G., Ferguson B., Varkey J., Park L.P., Breitschwerdt E.B., Woods C.W. (2014). Detection of *Bartonella* species in the blood of veterinarians and veterinary technicians: A newly recognized occupational hazard?. Vector-Borne Zoonotic Dis..

[85] Wormser G.P., Pritt B. (2015). Update and Commentary on Four Emerging Tick-Borne Infections: *Ehrlichia muris*-like Agent, *Borrelia miyamotoi*, Deer Tick Virus, Heartland Virus, and Whether Ticks Play a Role in Transmission of *Bartonella henselae*. Infect. Dis. Clin. N. Am..

[86] Leiby D.A. (2006). Babesiosis and blood transfusion: Flying under the radar. Vox Sang..

[87] Carpi G., Walter K.S., Mamoun C.B., Krause P.J., Kitchen A., Lepore T.J., Dwivedi A., Cornillot E., Caccone A., Diuk-Wasser M.A. (2016). *Babesia microti* from humans and ticks hold a genomic signature of strong population structure in the United States. BMC Genom..

[88] Lim P.L., Chavatte J.M., Vasoo S., Yang J. (2020). Imported Human Babesiosis, Singapore, 2018. Emerg. Infect. Dis..

