# Supplementary material for: Development of Multiplex Assays for the Identification of Zoonotic Babesia Species
Source: Pathogens. 2024 Dec 11;13(12):1094. doi: 10.3390/pathogens13121094 (PMC11676916; doi:10.3390/pathogens13121094)

Supplemental Material

**Table S1.** *Babesia* species currently identified by DNA sequence infecting people worldwide.

| Babesia sp | Gene | GenBank accession | Country | Reference |
|---|---|---|---|---|
| *B. bigemina* | 18S rRNA | OQ607820 | Ecuador | [4] |
| *B. bigemina* | 18S rRNA | MW227627 | Colombia | [25] |
| *B. crassa-like* | 18S rRNA | MW504968 | France | [26] |
| *B. crassa-like* | 18S rRNA | KX590751, KX590750 | China | [6] |
| *B. crassa-like* | 18S rRNA | MK240324 | Slovenia | [27] |
| *B. divergens* | 18S rRNA | KF533077 | Spain | [9] |
| *B. divergens* | 18S rRNA | LC167305 | Netherlands | [8] |
| *B. divergens* | 18S rRNA | MK510929 | Russia | [28] |
| *B. divergens* | 18S rRNA | Not available | France | [29] |
| *B. divergens* | 18S rRNA | HM355854 | China | [30] |
| *B. divergens* | 18S rRNA | AJ439713 | Portugal | [31] |
| *B. divergens* | 18S rRNA | GU945501 | Finland | [26] |
| *B. divergens* | 18S rRNA | MK256977 | China | [32] |
| *B. divergens-like* | 18S rRNA | AY274114 | USA | [33] |
| *B. duncani* | 18 SrRNA | HQ289870 | USA | [34] |
| *B. duncani* | 18S rRNA, ITS2 | AF158700, AF158701, AY027815, AY027816, AF15873, AF158705, AF158704, AY998763, AY998762, AY998761, AY965740, AY965741, AY998766, AY998765 | USA | [35] |
| *B. microti* | 18S rRNA | KT271759 | Spain | [36] |
| *B. microti* | suc | | USA | [37] |
| *B. microti* | CCTη, 18S rRNA | KT318131, KT318132, KT844553–KT844568 | Bolivia | [22] |
| *B. microti* | 18S rRNA | EF413181 | Germany | [38] |
| *B. microti* | 18S rRNA | Not reported | Denmark | [39] |
| *B. microti* | 18S rRNA | KT429729 | Poland | [40] |
| *B. microti* | 18S rRNA | KJ783438 | Poland | [41] |
| *B. microti* | *coxA*, BMN1-11 | MW665112-MW665117 | USA | [21] |
| *B. microti* | 18S rRNA | MZ727029 | USA | [42, 43] |
| *B. microti* | 18S rRNA, *cox1*, ITS | MK609547, MK609548 | USA | [32] |
| *B. microti* | 18S rRNA | MH048838 | Mexico | [44] |
| *B. microti* | ITS1 | GU230755 | Nicaragua | [31] |

| | | | | |
|---|---|---|---|---|
| *B. microti* | 18S rRNA, *β-tubulin*, CCT-eta | LC314654- LC314658, LC314659-LC314663, LC314664-LC314668 | USA | [45] |
| *B. microti* | 18S rRNA, β-tubulin | JX417370, JX417371 | Australia | [46] |
| *B. microti* | 18S rRNA | Not available | USA | [47] |
| *B. microti* | 18S rRNA | KC470047 | Poland | [48] |
| *B. microti* | 18S rRNA, β-tubulin | KF410825, KF410827, KF410824, KF410826, KJ128386-KJ128388 | China | [49] |
| *B. microti* | 18S rRNA | KF410824 - KF410827 | China | [50] |
| *B. microti-like* | 18S rRNA | AB032434 | Japan | [1] |
| *B. odocoilei* | 18S rRNA | MW368483, MW368482 | Canada | [28] |
| *B. venatorum* | 18S rRNA | AY046575 | Italy, Austria | [58] |
| *B. venatorum* | 18S rRNA, β-tubulin | OP522105, OP559478 | China | [51] |
| *B. venatorum* | 18S rRNA | KM244044 | China | [52] |
| *B. venatorum* | 18S rRNA | KF724377 | China | [53] |
| *B. motasi* | 18S rRNA, Cytochrome b, cox3 | MK930513, MK918505, MK918507 | USA | [54] |
| *Babesia* sp. EU3 | 18S rRNA | Not available | Germany | [55] |
| *Babesia* sp. FR1 | 18S rRNA, ama-1, rap-1 | MZ825347, MZ836261, MZ836260 | France | [56] |
| *Babesia* sp. KO1 | 18S rRNA | DQ346955 | Korea | [43] |
| *Babesia* sp. XXB | 18S rRNA | KU291357 | China | [57] |

**Table S2.** Piroplasmida species, animal source, case identification and GenBank Accession numbers generated in this study for the intergenic transcribed spacer regions between 18S rRNA and 5.8S rRNA (ITS1) and 5.8S rRNA and 28S rRNA (ITS2). Note: N/A: not amplified.

| **sp.** | **Host** | **Case ID** | **ITS1** | **GenBank Accession number** | **ITS2** | **GenBank Accession number** |
|---|---|---|---|---|---|---|
| *Babesia canis* | Dog | VB20-04614 | **Yes** | PQ404823 | **Yes** | PQ404832 |
| *Babesia capreoli* | *Ixodes ricinus* | UKHSA | **Yes** | PQ404828 | **Yes** | PQ404838 |
| *Babesia* sp. Coco | Dog | VB21-00891 | **Yes** | PQ415085 | **Yes** | PQ415090 |
| *Babesia conradae* | Dog | VB23-011673 | No | N/A | **Yes** | PQ404842 |
| *Babesia divergens* | Rabbit | MO-1 | **Yes** | PQ404827 | **Yes** | PQ404837 |
| *Babesia duncani* | Hamster | WA-1 | **Yes** | PQ415093 | No | N/A |
| *Babesia felis* | Cat | VB17-02733 | **Yes** | PQ415084 | **Yes** | PQ415089 |
| *Babesia gibsoni* | Dog | VB20-03041 | **Yes** | PQ404826 | **Yes** | PQ404836 |
| *Babesia gibsoni* | Dog | VB20-05838 | **Yes** | PQ404830 | **Yes** | PQ404840 |
| *Babesia lengau* | Cat | VB18-01695 | **Yes** | PQ415083 | **Yes** | PQ404835 |
| *Babesia microti* | Hamster | GI-1 | **Yes** | PQ404825 | **Yes** | PQ404834 |
| *Babesia negevi* | Dog | R2024a | **Yes** | PQ415086 | **Yes** | PQ415091 |
| *Babesia odocoilei* | Caribou | VB19-09086 | **Yes** | PQ404843 | **Yes** | PQ411178 |
| *Babesia odocoilei* | Reindeer | VB19-09287 | **Yes** | PQ404844 | **Yes** | PQ411179 |
| *Babesia odocoilei* | Reindeer | VB19-09670 | **Yes** | PQ404845 | **Yes** | PQ411180 |
| *Babesia vogeli* | Dog | VB21-01537 | **Yes** | PQ404824 | **Yes** | PQ404833 |
| *Babesia vogeli* | Dog | R2024b | **Yes** | PQ404829 | **Yes** | PQ404839 |
| *Babesia vulpes* | Dog | VB21-010325 | **Yes** | PQ415082 | **Yes** | PQ415088 |
| *Cytauxzoon felis* | Cat | VB20-03823 | **Yes** | PQ404831 | **Yes** | PQ404841 |
| *Theileria bicornis* | Rhinoceros | VB19-09934 | **Yes** | PQ415087 | **Yes** | PQ415092 |

**Table S3.** Assessment of *Babesia* species specificity and cross-amplification with other *Babesia* species for the species-specific ITS1 assay. Note: original refers to DNA extracted from blood from pre-characterized animal clinical samples.

| | | | qPCR specific assays (Cq) | | | |
|---|---|---|---|---|---|---|
| **Piroplasma sp.** | **Case ID** | **Samples** | ***B. odocoilei*** | ***B. divergens*** | ***B. duncani*** | ***B. microti*** |
| *B. odocoilei* | VB19-09386 | Original | 23.5 | Neg | Neg | Neg |
| *B. odocoilei* | VB19-09386 | Diluted 1:10 | 27.1 | Neg | Neg | Neg |
| *B. odocoilei* | VB19-09386 | Diluted 1:100 | 31.0 | Neg | Neg | Neg |
| *B. divergens* | R-1 | Diluted 1:100 | Neg | 33.9 | Neg | Neg |
| *B. divergens* | R-1 | Diluted 1:1000 | Neg | 36.9 | Neg | Neg |
| *B. duncani* | WA-1 | Original | Neg | Neg | 26.9 | Neg |
| *B. duncani* | WA-1 | Diluted 1:10 | Neg | Neg | 31.0 | Neg |
| *B. duncani* | WA-1 | Diluted 1:100 | Neg | Neg | 35.6 | Neg |
| *B. duncani* | WA-1 | Diluted 1:1000 | Neg | Neg | 39.3 | Neg |
| *B. microti* | GI-2 | Original | Neg | Neg | Neg | 26.0 |
| *B. microti* | GI-2 | Diluted 1:10 | Neg | Neg | Neg | 24.5 |
| *B. microti* | GI-2 | Diluted 1:100 | Neg | Neg | Neg | 37.3 |
| *B. microti* | GI-2 | Diluted 1:1000 | Neg | Neg | Neg | 33.9 |
| *B. canis* | VB18-07290 | Original | Neg | Neg | Neg | Neg |
| *B. canis* | VB20-01614 | Original | Neg | Neg | Neg | Neg |
| *B.* coco | VB20-06987 | Original | Neg | Neg | Neg | Neg |
| *B.* coco | VB17-04969 | Original | Neg | Neg | Neg | Neg |
| *B. vulpes* | VB21-010325 | Original | Neg | Neg | Neg | Neg |
| *B. vulpes* | VB21-01000 | Original | Neg | Neg | Neg | Neg |
| *B. vulpes* | VB22-003333 | Original | Neg | Neg | Neg | Neg |
| *B. vulpes* | VB22-008037 | Original | Neg | Neg | Neg | Neg |
| *B. conradae* | VB23-011673 | Diluted 1:100 | Neg | Neg | Neg | Neg |
| *B. lengau* | VB18-01695 | Original | Neg | Neg | Neg | Neg |
| *B. gibsoni* | VB20-08351 | Original | Neg | Neg | Neg | Neg |
| *B. gibsoni* | VB20-05838 | Original | Neg | Neg | Neg | Neg |

## Supplementary Material 5

In preliminary studies, DNA samples were extracted from deer blood and analyzed. First, PCR tests targeting the 18S rRNA gene were conducted. During sequence analysis, a significant number of overlapping peaks were observed, suggesting possible co-infection (Figure S1). The sequences were then trimmed, and the cleanest portion exhibited 99.73% identity with *Theileria cervi* by BLASTn.

**Figure S1.** Electropherogram of an 18S rRNA gene sequence showing the overlapping peaks.

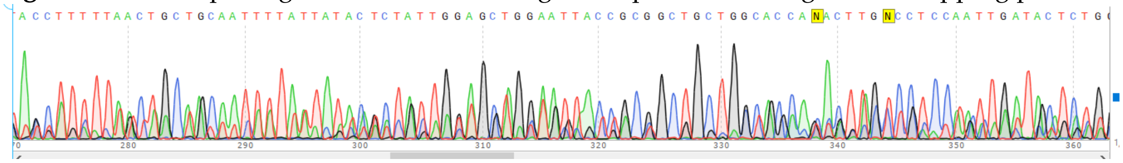

A specific qPCR assay targeting the ITS-1 region of *B. odocoilei* was performed, as described in this manuscript. Some animals tested positive, including one that had a sequence similar to *T. cervi* in the 18S rRNA gene (here named as Deer 4). The PCR product from this animal was sequenced, and the sequence was identical to *B. odocoilei*.

An alignment comparing the 18S rRNA sequences of *T. cervi*, *B. odocoilei*, and the deer samples revealed that the sequences from the deer were indeed closer to *T. cervi* (Figure S2). In contrast, the alignment based on the ITS-1 intergenic region confirmed the presence of *B. odocoilei* (Figure S3).

**Figure S2.** Part of the 18S rRNA alignment of *T. cervi*, *B. odocoilei* and deer sequences. Showing great divergence between *T. cervi* and *B. odocoilei*.

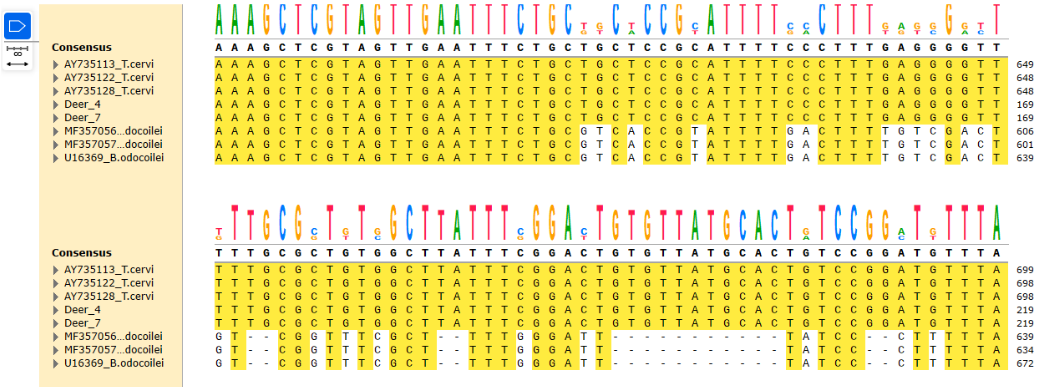

**Figure S3.** Alignment of ITS-1 sequences.

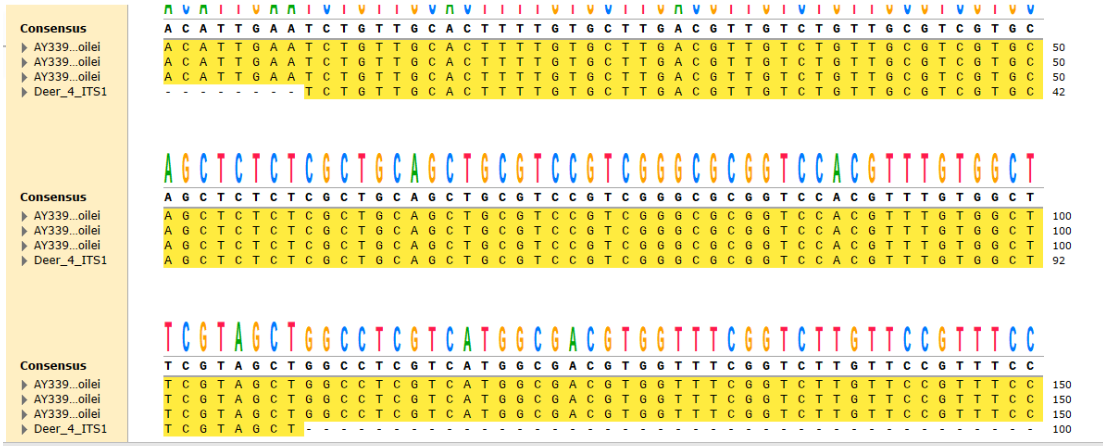

Supplement: Supplementary file 1 [file pathogens-13-01094-s001.zip › pathogens-3326679-supplementary.pdf]
